# Editorial to Special Issue—Composition and Biological Properties of Bee Products

**DOI:** 10.3390/foods11040608

**Published:** 2022-02-20

**Authors:** Maria Graça Miguel

**Affiliations:** Mediterranean Institute for Agriculture, Environment and Development (MED), Faculdade de Ciências e Tecnologia, Departamento de Química e Farmácia, Universidade do Algarve, Campus de Gambelas, 8005-139 Faro, Portugal; mgmiguel@ualg.pt

Honey continues to be the most studied bee product, with 60% of articles addressing this thematic [1,2,3,4,5,6] and, more specifically, its biological properties [1,2,3,4]. Only one article was focused on the effect of heavy metals, pesticides, and antibiotic residues on the quality of honey [5], and only one article had the honey of the African stingless bee *Meliponula ferruginea* as its study objective [6]. Bee pollen [7,8], cerumen [6], royal jelly and drone brood [9], and propolis [6,10] were also addressed and, once again, with studies focusing on the biological properties [6,8,9,10].

Scripcă and Amarici [2] studied the effect of heavy metals, pesticides, and antibiotic residues on the quality of honey. For this study, the authors [2] used the following Romanian honeys: black locust (*Robinia pseudoacacia*), rapeseed (*Brassica napus*), dandelion (*Taraxacum officinale*), mint (*Mentha spicata*), sunflower (*Helianthus annuus*), buckwheat (*Fagopyrum esculentum*), raspberry (*Rubus idaeus*), and meadow honey. Despite the ban of antibiotic residues in honey, the results obtained showed that antibiotic residue incidence was 25%, with nitrofurantoin present at the highest concentration in mint honey (5.528 ng/g). In contrast, meadow honeys were free from antibiotics, and the lowest antibiotic concentration (0.2 ng/g) was found in raspberry and black locust honeys. The lowest pesticide content was also found in raspberry and meadow honeys, maybe because they are coming from wild areas without the pressure of pesticide and fertilizer utilization in crops. Pesticide residues were found in 68% of samples, but, in all cases, the presence of these pesticide residues was lower than the maximum residue limits (MRLs) required by European legislation.

Antioxidant [1,2,4], antimicrobial [2,3], and anti-inflammatory activities [2] were also reported for honey samples from diverse geographical and botanical origins. Boutoub et al. [4] reported that the capacity of seven honey samples of *Euphorbia* origin from Morocco for scavenging the free radicals nitric oxide (^•^NO), superoxide anion radicals (O^•−^), and 2,2-diphenyl-1-picrylhydrazyl (DPPH) could be attributed to the phenolic fraction since they presented better activity after separating these secondary metabolites from the honey. In contrast, Alevia et al. [2] found higher antioxidant activity of raw honeys from Spain than the methanolic extracts. Moreover, the digests also presented higher antioxidant activity than the extracts. The inhibition of several enzyme (acetylcholinesterase, tyrosinase, lipoxygenase, xanthine oxidase, and hyaluronidase) activities was also evaluated in Moroccan [4] and Spanish honeys, respectively [2], as well as in the respective aqueous or methanolic extracts, and, in both cases, the activities were better in the extracts. Starowicz et al. [1] demonstrated a relationship between the browning index, color, total phenolics, and antioxidant activity of monofloral (acacia, buckwheat, heather, and linden) and multifloral honeys from Poland. The results obtained permitted establishing a correlation between the brown pigments and the antioxidant activity. They also demonstrated that the brown pigments appear after a Maillard reaction and melanoidin formation, even in unheated Polish-originated honeys. Much interesting was the link found by the authors [1] between the total phenols and the formation of melanoidins in Polish honeys.

All Spanish samples (honeys and digested honeys) showed antimicrobial activity against *Staphylococcus aureus*, *Listeria monocytogenes*, *Escherichia coli*, *Streptococcus mutans*, *Pseudomona aeruginosa*, and one yeast (*Candida albicans*). Nevertheless, the digested honeys showed higher antimicrobial activity against *S. aureus* and *L. monocytogenes*. According to these results, the authors [2] pointed out the positive role of honey in the human body after the digestion process. On the other hand, the methanolic extracts (obtained from honeys) could be used in cosmetics and pharmaceuticals due to their higher anti-hyaluronidase activity in comparison to the raw honeys.

Currently, the resistance of microorganisms to antibiotics is rising and constitutes a major health problem. This resistance seems to be correlated to the formation of biofilm by bacteria [3]. For this reason, the authors investigated the antibacterial and antibiofilm activity of three Hungarian monofloral honeys (black locust, linden, and sunflower) against common respiratory tract pathogens, including the biofilm-forming *Haemophilus* spp., *P. aeruginosa*, and *S. pneumoniae*. All honey samples at the MIC/2 concentration were able to inhibit the formation of biofilm by each bacterium strain tested, linden honey being the most effective and the black locust honey being the least. Propolis and cerumen of the African *Meliponula ferruginea* from Tanzania (Kilimanjaro, Ngarony locality) also had antimicrobial activity against *Staphylococcus aureus* ATCC 25923, *Enterococcus faecalis* ATCC 29212, *Listeria monocytogenes* ATCC 7644, *Pseudomonas aeruginosa* ATCC 27853, *Salmonella typhi* ATCC 14028, *Escherichia coli* ATCC 25922, and *Candida albicans* ATCC 10239, as well as anti-biofilm activity at MIC levels (53–89.0% of inhibition) [6]. Such results are promising as they will provide another source of income for farmers in less favored regions. According to the authors [6], propolis and cerumen also had anti-quoring sensing properties but at different MIC and sub-MICs. Concerning the chemical composition of honey, propolis and cerumen of the African *Meliponula ferruginea*, the authors considered trehalulose as a potential marker for African stingless bee honey. Concerning propolis and cerumen, di- and triterpenoids dominated, although with qualitative and quantitative differences among samples.

Only one work about bee pollen has its discrimination spectroscopic identification as the sole target. In this context, Bleha et al. [7], in a communication paper, hypothesized that the difference in composition and color, in connection with the botanical source, would allow discriminating bee pollen by spectroscopic identification. Thus, the authors [7] concluded that a combination of spectroscopic imaging (Fourier transform (FT) mid- and near-infrared (FT-MIR, FT-NIR), and FT-Raman spectra) of bee pollen and statistical methods using principal component analysis (PCA) were good tools for evaluating the composition and quality of eleven bee pollen samples from diverse localities and regions of the Slovak Republic, with the advantage of being non-destructive. Moreover, FT-Raman spectra also enabled indicating plant pigments as chemical markers of botanical origin. Nevertheless, the authors [7] claim that a proper FT-IR/Raman bee pollen library for reference use is needed, which needs to be exploited and developed in the near future.

The biological effects of bee products, such as pollen samples, are related to the content and composition of polyphenol compounds (hydroxycinnamic acid and flavonol glycosides) and carotenoids, although many other compounds can be found (carbohydrates, proteins, enzymes, cofactors, unsaturated and saturated fatty acids, minerals, trace elements, essential amino acids, and vitamins, especially B, A, C, and E), that exert antioxidant, anti-inflammatory, antimicrobial, antifungal, anti-mutagenic, and antitumor effects [8]. The authors [8] wanted to complement their data of in vitro antioxidant activities of polyfloral (*Castanea*, *Rubus*, and *Cistus*) Tuscan bee pollen previously determined. For this purpose, they added the results obtained from ex vivo biological tests using human erythrocytes to the existing information. The presence of bee pollen samples of *Castanea* and *Cistus* origins were able to prevent lipid peroxidation and hemolysis, thus being good free radical scavengers. The anti-hemolytic activity of samples was even better than trolox, the standard used for comparison. In addition, the authors [8] investigated, for the first time, the protective effect of *Castanea* bee pollen in human microvascular endothelial cells (HMEC-1) under endoplasmic reticulum stress conditions induced by thapsigargin. The authors [8] found that 10 ng/mL of *Castanea* bee pollen was able to reduce gene overproduction (C/EBP-homologous protein (CHOP), cyclooxygenase-2 (COX-2), intercellular adhesion molecule 1 (ICAM-1)) and oxidation processes. In contrast, the antimicrobial activity evaluated by the authors against *Escherichia coli*, *Salmonella typhimurium*, *Enterobacter erogene*, *Enterococcus faecalis*, and *Staphylococcus aureus* was poor.

Drone brood (DB) is collected from beehives as young male larvae, usually between days 4 and 14 of development. DB and royal jelly (RJ) are both yellow, and the former is sometimes used to counterfeit RJ since it can be easily obtained and in larger quantities than RJ [9]. For the first time, the authors [9] conducted a detailed study on the chemical composition of DB at various stages of development and RJ that originated from the same apiaries for comparison. This approach would permit finding the specific markers for RJ and DB in order to distinguish them. Despite the similarities between these two bee products, DB was richer in iron and manganese and reduced more sugars and amino acids (tyrosine, proline, and leucine) than RJ. Diastase and α-glucosidase activity as well as ferulic and ellagic acids were only found in DB. It also contained more testosterone on the 14th day and estradiol on the 7th day than RJ. Since RJ is poorer in hormonal activity than DB, this could be a way to detect the adulteration of RJ with DB [9].

A review on the biological properties of propolis, particularly antibacterial, antiviral, antifungal, and antiparasitic activities, as well as the human clinical trials developed so far, was carried out by Zulhendri et al. [10], demonstrating that propolis is effective and safe as an adjuvant therapy to combat infections. Moreover, the mechanisms involved in these activities involving the pathogens and observed in the host were also reviewed and discussed. Propolis acts on the pathogens through several mechanisms: by forming a physical barrier between the pathogen and the host; by inhibiting the enzymes and proteins needed for invading the host cells; by inhibiting the replication of pathogens; by inhibiting the metabolic pathways of pathogens through the disruption of cellular organelles and components indispensable for the production of energy [10]. In what concerns the host, propolis upregulates the innate immunity, modulating the inflammatory signaling pathways as well as the oxidative balance.

These articles extended the knowledge on bee products by:

− finding easier methods to discriminate honey;
− finding possible markers for royal jelly and drone brood in order to prevent the royal jelly counterfeit;
− finding possible markers for honey, propolis, and cerumen of the African *Meliponula ferruginea*;
− finding the factors that contribute to the antimicrobial, antioxidant, and anti-inflammatory properties of honey from different floral and geographical origins;
− finding the effect of heavy metals, pesticides, and antibiotic residues on the quality of Romanian honeys;
− finding the mechanisms involved in antibacterial, antiviral, antifungal, and antiparasitic activities of propolis.
